# Biological significance of C-reactive protein, the ancient acute phase functionary

**DOI:** 10.3389/fimmu.2023.1238411

**Published:** 2023-10-04

**Authors:** Shelley Bhattacharya, Chayan Munshi

**Affiliations:** ^1^ Department of Zoology, Visva-Bharati University, Santiniketan, India; ^2^ Ethophilia (An Autonomous Research Group), Santiniketan, India

**Keywords:** C-reactive protein, acute phase response, acute phase protein, innate immunity, inflammatory

## Abstract

C-reactive protein (CRP) is one of the major members of the family of acute phase proteins (APP). Interest in this CRP was the result of a seminal discovery of its pattern of response to pneumococcal infection in humans. CRP has the unique property of reacting with phosphocholine-containing substances, such as pneumococcal C-polysaccharide, in the presence of Ca^2+^. The attention regarding the origin of CRP and its multifunctionality has gripped researchers for several decades. The reason can be traced to the integrated evolution of CRP in the animal kingdom. CRP has been unequivocally listed as a key indicator of infectious and inflammatory diseases including autoimmune diseases. The first occurrence of CRP in the evolutionary ladder appeared in arthropods followed by molluscs and much later in the chordates. The biological significance of CRP has been established in the animal kingdom starting from invertebrates. Interestingly, the site of synthesis of CRP is mainly the liver in vertebrates, while in invertebrates it is located in diverse tissues. CRP is a multifunctional player in the scenario of innate immunity. CRP acts as an opsonin in the area of complement activation and phagocytosis. Interestingly, CRP upregulates and downregulates both cytokine production and chemotaxis. Considering various studies of CRP in humans and non-human animals, it has been logically proposed that CRP plays a common role in animals. CRP also interacts with Fcγ receptors and triggers the inflammatory response of macrophages. CRP in other animals such as primates, fish, echinoderms, arthropods, and molluscs has also been studied in some detail which establishes the evolutionary significance of CRP. In mammals, the increase in CRP levels is an induced response to inflammation or trauma; interestingly, in arthropods and molluscs, CRP is constitutively expressed and represents a major component of their hemolymph. Investigations into the primary structure of CRP from various species revealed the overall relatedness between vertebrate and invertebrate CRP. Invertebrates lack an acquired immune response; they are therefore dependent on the multifunctional role of CRP leading to the evolutionary success of the invertebrate phyla.

## Phylogenetic history of CRP

Because of its special ability to react with the C-polysaccharide found in the pneumococcal cell wall, the protein is known as C-reactive protein (CRP) (1). Significant studies on CRP from *Limulus polyphemus* in the last century have conclusively proven the substance’s antiquity (2, 3). CRP synthesis is activated in mammals (e.g., humans and rabbits), but it is constitutively expressed in the arthropod *Limulus* and makes up a significant portion of the hemolymph of horseshoe crabs at a concentration of 1-2 mg/mL (3). Additionally, the ophuirid (echinodermata) *Ophiocomina nigra* was found to have the CRP gene (4). A great deal of interest has been generated in investigating the relationships between the CRP gene and thromboxane A2, another chemical expressed in ophiurids, due to the properties of the transcriptome CRP that were determined to be particularly significant in this species (5, 6).

CRP has also been identified in the large African snail *Achatina fulica* (ACRP) as a typical hemolymph component (7). The newly hatched male has a concentration of 1 mg/mL, the most active hermaphrodite has a concentration of 3-5 mg/mL, and the sedentary female has a concentration of 1.5-2.8 mg/mL, demonstrating a direct association between the protein and the active phase of the animal (8). Like other vertebrate CRP, ACRP also acts as a scavenger of chromatin fragments as evidenced by its binding to the polycation poly-L-arginine. It is interesting that ACRP was found to enhance rat platelet aggregation *in vitro* in the presence of ADP and Ca^2+^, suggesting a probable role of ACRP in the aggregation of amoebocytes during the formation of plugs in injured tissue.

The estimated molecular weight of ACRP is 400 kDa, with a high absorbance in the 200–230 nm range, and it was discovered to be made up of four subunits with respective molecular weights of 110, 90, 62, and 60 kDa (8). The metal binding protein metallothionein, whose level in the hemolymph of *Achatina* increases from the male to the hermaphrodite to the female, showing a pattern that is very different from that of the ACRP titer, has been observed in vertebrates. ACRP has a high molar ratio (five) of metal binding, demonstrating its ability to sequester heavy metals. It was determined that the high level of metallothionein compensates functionally for the low titer of ACRP since both metallothionein and ACRP can sequester inorganic mercury in the sedentary female (8).

CRP has been observed in animals other than humans, including the freshwater fish *Channa punctatus* (9) and the Nile tilapia *Oreochromis niloticus* (10). Based on amino acid sequence analysis and the number of subunits, the main structure of human and rabbit CRP has been identified. CRP has been categorized into the pentraxin family of proteins (11, 12). As a result, gene sequence analyses provide information about the structure–function relationship of *Limulus* CRP as well as strong evidence of the two species’ overall sequence similarity.

CRP is a cyclic oligomer found in different species that consists of nearly similar subunits with molecular weights of 20–30 kDa. Phosphocholine is bound by CRP in a Ca^2+^-dependent way. It has also been shown that CRP from different species exhibit immunological cross-reactivity (13). It has been demonstrated that CRP exhibits ligand-binding specificities dependent on structure. Further research on invertebrate CRP is necessary to understand the factors that led to this evolution of CRP, as it has been suggested (13) that changes in intra- and inter-chain disulfide bonds and the glycosylation status of CRP caused the different structure–function relationships in various species.

The physiological role of CRP has not been well studied, despite a large number of findings on its biological effects in model systems and *in vitro*. Mice with endogenous CRP levels that are low even after an inflammatory stimulus were reported to be protected against infections with *Bacillus subtilis* and *Salmonella typhimurium* by *Achatina* CRP (ACRP). In addition, it was noted that the bacteria’s growth curves demonstrated that ACRP has opposing effects on the two different types of bacteria, acting at a concentration of 50 μg/mL to be bacteriostatic against gram-negative salmonellae and bactericidal against gram-positive bacilli (14).

It has been established that the mechanism of action of ACRP was mediated by a loss of energy in the bacterial cells where ACRP inhibits the important carbohydrate metabolizing enzymes, disturbs the cellular redox potential homeostasis, reduces glutathione status, and is associated with a significantly increased rate of lipid peroxidation (14). By activating poly (ADP-ribose) polymerase-1 and caspase-3, ACRP may also cause the bacterial cells to die in a manner similar to apoptosis. Therefore, the production of reactive oxygen species (ROS) and metabolic impairment that resulted in apoptosis were the causes of the bacterial cells’ demise (14). ACRP also works to reduce the load of environmental contaminants like lead (15). In this case, CRP was used to treat lead-treated mice and rats and reverse the hepatotoxicity (15).

*Achatina fulica* has attained widespread distribution and is currently regarded as one of the most successful evolutionary creatures. Researchers have been working to understand their intricate immune system over the past few decades in order to collect useful chemicals to treat human diseases. It has been shown that *Achatina* has crucial immunological components such the coagulation system, innate immune molecules, bioactive proteins, and CRP (16). The evolutionary importance of *Achatina* having strong innate immune defences and infection-fighting abilities has also been noted (17).

## Functional multiplicity of CRP

CRP is a versatile protein that has been known about since 1982 (18). It has long been known that vertebrates produce CRP as an acute phase protein (APP). CRP is a constitutively active protein in the hemolymph of the invertebrate phyla Arthropoda, Echinodermata, and Mollusca, whereas in the serum of vertebrates the concentration of CRP increases significantly following bacterial infections or other triggers such environmental toxins (19).

## Fish CRP

Five genes encoding similar molecules to CRP/serum amyloid-P component (SAP) were found when the entire genome of the Atlantic salmon (*Salmo salar*) was analyzed (20). It is interesting to note that these genes were divided into Group I (CRP/SAP-1a, CRP/SAP-1b, CRP/SAP-1c, and CRP/SAP-2) and Group II (CRP/SAP-3). The first group, known as the universal group, is found in all vertebrates, whereas the second group is unique to fish and amphibians. CRP/SAP-1a were found to be elevated by the cytokines interleukin (IL)- and interferon (IFN) in head kidney cells; however, the other four CRP/SAP were resistant, according to gene expression analyses (20). Serrum amyloid-A5 (SAA-5) was the main APP in salmon, whose expression was solely induced by *Aeromonas salmonicida* in Atlantic salmon. This finding illustrates the potential functional distinction between salmon CRP/SAP and its mammalian homologues.

Nile tilapia’s CRP gene has also been discovered (10). The data clearly demonstrate that CRP in Nile tilapia is a robust and active participant in the anti-bacterial immune response beginning with the agglutination of bacteria as well as the regulation of phagocytosis and inflammation. Tilapia CRP also plays a function during bacterial infection. Future research in this area will shed light on the defences the fish CRP employs against bacterial invasion.

## CRP in mammals

Under metal stress, the rabbit experiences a number of alterations, including the occurrence of CRP in the blood and a notable decrease in the serum titers of albumin and acetylcholinesterase (19). Marine mammals have also been linked to APP (21). The serum of manatees was used in immunological cross-reactivity experiments using SAA, haptoglobin, 1-acid glycoprotein, and CRP, and it was discovered that CRP was not a significant APP in this species (21). According to reports, SAA has this species’ best diagnostic sensitivity for inflammatory illness (21). In stressed and injured manatees, SAA has been discovered to be an important prognostic marker (22). Additionally, specific CRP assays have been developed for use with serum samples from harbor seals suffering from inflammation-related illnesses such as pneumonia (23).

Several species of filarial worms are responsible for canine filariasis (24). The filaria lifecycle is primarily responsible for the pathophysiological response to infection. Many animal diseases, including filariasis in dogs afflicted with *Dirofilaria immitis*, *Brugia pahangi*, or both parasites, are diagnosed using serum protein profiles and CRP levels. Dogs with *D. immitis* or *B. pahangi* infections had average CRP levels of 69.9 mg/L and 12.9 mg/L, respectively. In contrast to *B. pahangi*-infected dogs, those with *D. immitis* infections had abnormally high CRP concentrations (24).

## Human CRP

Abdominal aortic aneurysm (AAA) and the CRP polymorphism rs3091244 are related. According to reports, AAA involves an inflammatory process whose modulation is under the control of CRP (25). In a case–control study with two distinct populations of AAA patients, it was found that the rare T and A alleles were significantly associated with AAA presence in both populations and correlated with higher CRP levels and AAA diameter. This conclusion was drawn from the frequency of the functional triallelic (C, T, and A alleles) rs3091244 polymorphism.

The existing global data allow for the conclusion that atherosclerosis is the primary pathophysiologic contributor to cardiovascular disease, which is the major cause of morbidity and mortality in the adult population (26). The involvement of CRP in atherosclerosis is highlighted (26) because it is a fundamental aspect of chronic inflammation, is highly resistant to proteolysis, and is primarily synthesized in the liver in response to proinflammatory cytokines like IL-6, IL-1β, and tumor necrosis factor (TNF). In addition to causing apoptosis, vascular cell activation, monocyte recruitment, lipid buildup, and thrombosis, CRP also directly stimulates the complement system (26). It is interesting to note that in peripheral tissue and atheromatous plaques, where each form exhibits distinct affinities for ligands and receptors and exerts different effects in the progression of atherosclerosis, CRP dissociates from its native pentameric form into a monomeric form, and it was determined that CRP is a reliable criterion for the assessment of cardiovascular risk (26).

In one investigation, New Zealand white rabbits were used to assess how immunization affects APP (27). Plasma CRP levels changed after receiving several experimental vaccinations (27). When rabbits were treated with vaccines containing novel adjuvants that activate Toll-like receptors, it was observed that the incidence and intensity of responses associated with the acute phase response (APR), both positive and negative APP, increased. The notable changes in the plasma levels of CRP served as a foundation for the proposal of a classification scheme of high, medium, low, and none. According to the study’s findings, the alterations in plasma proteins show that systemic inflammation is becoming more severe and is associated with significant clinical adverse effects in addition to reflecting an activation of the APR (27).

Erythrocyte sedimentation rate and CRP were measured in patients with hepato-splenomegaly, high blood pressure, diabetic mellitus with polyneuropathy, oral cavity infection, and other conditions of unknown etiology (28). The erythrocyte sedimentation rate was shown to be less sensitive and accurate in reflecting the APR than the CRP (28). With detection limits of less than 0.3 mg/L and a stronger risk prediction than LDL cholesterol, CRP levels were very high in arthritis and were thought to be positively associated with the risk of future coronary events like coronary artery, cerebrovascular, and peripheral arterial diseases (28). Nevertheless, in such cases, the use of CRP assay is advised.

In patients with hyperleptinemia, the interaction of CRP with the leptin receptor has also been documented (29). Ironically, leptin insufficiency is the root cause of morbid obesity, and people with this condition have elevated leptin levels. CRP is crucial in this medical condition for binding to leptin. The study of blood levels of CRP, leptin, and soluble leptin receptor following a solid phase binding test and the co-immunoprecipitation of CRP and soluble leptin receptor from human plasma with elevated levels of CRP were used to validate the interaction of CRP (29).

CRP is known to be largely produced by hepatocytes and can increase by a factor of 1,000 at infection sites. The native form of CRP is a homopentameric protein that permanently splits into five monomeric forms of CRP. However, smooth muscle cells, macrophages, endothelial cells, lymphocytes, and adipocytes are also capable of producing CRP. The CRP levels may also be impacted by estrogen used in hormone replacement therapy (30).

CRP can be found in two different states—native pentameric and monomeric—and these two forms can attach to various receptors or lipid rafts to take on various functional characteristics. While some studies have shown that CRP is also linked to chronic inflammation, it is recognized as a biomarker of acute inflammation. This indicates the clinical importance of CRP in chronic inflammatory and neurodegenerative diseases, including the role of CRP and its forms specifically in the pathogenesis of these diseases, including cardiovascular disease, type 2 diabetes mellitus, age-related macular degeneration, hemorrhagic stroke, Alzheimer’s disease, and Parkinson’s disease. These developments need to be translated into reliable methods for the diagnosis and treatment of inflammatory diseases (31).

It is commonly acknowledged that CRP has both pro- and anti-inflammatory characteristics. By binding to phosphocholine, phospholipids, histone, chromatin, and fibronectin, CRP plays a significant role in the identification and removal of pathogens as well as injured cells. It has been found to speed up the elimination of cellular debris, injured or apoptotic cells, and foreign pathogens by activating the traditional complement system and the phagocytic cells via Fc receptors (32). As seen in idiopathic thrombocytopenic purpura (32), this turns pathogenic if activated by autoantibodies with the phosphocholine arm that cause auto-immune processes. The indirect test of ESR for inflammation, on the other hand, has revealed that CRP cannot distinguish between bacterial and non-bacterial illnesses, and that CRP levels rise quickly at the start of an inflammatory stimulus and fall when it is removed (33–35). Elevated CRP levels are persistent in conditions like chronic inflammation or rheumatoid arthritis. Such elevated CRP levels have been observed in both acute and chronic diseases, with either infectious or non-infectious etiologies. However, noticeably increased levels of CRP are frequently connected to pathogen- or infection-related molecular pattern recognition (36). CRP levels increase with trauma (an alarm reaction), but these elevations have also been linked to a variety of conditions, from sleep disorders to periodontal disease. It can be challenging to draw any conclusions regarding the importance of a high CRP level as a prognostic marker for cardiovascular disease because chronic diseases such inflammatory arthritis or SLE can result in chronically elevated CRP (32). As a result, it was suggested that CRP levels above 50 mg/dL are typically caused by a high rate of bacterial infection and have been used as a prognostic indicator for both acute and chronic cases of hepatitis C, dengue fever, and malaria (37–39).

## Concluding remarks

Animals have two different types of response proteins: heat shock proteins and APP. The end result of a long-ago stress reaction to many sorts of stress-inducing environmental stressors are heat shock proteins. It has been noted that biodiversity stress responses are brought on by changing conditions and are significantly associated to the local biotic and abiotic components (40). Furthermore, it is an inducible response at a level probably determined during their development in a temperature-labile environment (41). The tolerance range of environmental temperature, on the other hand, reveals the resistance to climate change (42). Contrarily, a general systemic response is elicited in response to acute circumstances of inflammation brought on by primarily bacterial exposure, and specific proteins are categorized under acute phase proteins, where they are distinguished as CRP brought on by tissue damage (43). Since air-breathing animals, both invertebrates and vertebrates, have retained the imprint of heat shock response proteins during their history, it may be inferred that the evolution of CRP is controlled by epigenetic responses acquired during that time.

In general, all animals respond to all types of wounds and stress to keep the body’s homeostasis system in place. This equilibrium is either achieved by specific or by non-specific mechanisms, such as leukocytosis as well as cytological and cytokine reactions. These reactions—the APR—lead to an alteration in the serum concentration of acute phase proteins. It has recently been proven that such assessments of the serum concentration of these acute phase proteins aid in determining the health condition of animals and disease prognosis. Since the APR is a dynamic process, when the triggering element is absent, the serum concentration of the APRs recovers to the basal level. Additionally, the APRs cause metabolic changes and send out early, non-specific signals of defence against an insult before specific immunity is developed. Therefore, there is great potential for CRP to be used in contemporary veterinary practise for illness diagnosis and health status evaluation in animals (44).

Recent research has shown that major depressive disorder in humans negatively affects the mental health of 264 million people worldwide (45). Although the risk has not been tied to polygenic CRP, blood CRP level has been found to be strongly associated with a history of mental disorders (46). Studies have also shown that polymorphisms in the CRP gene might have directly altered production of CRP in the liver in these patients (47). There is evidence that people with major depressive disorder have higher levels of CRP and other proinflammatory biomarkers.

Researchers have discovered a novel function for CRP, eloquently demonstrating the evolutionary significance of this old molecule. CRP initially manifested in invertebrates as constitutively produced proteins without pro- or anti-inflammatory characteristics. The intricate roles of CRP have gradually come into focus in higher evolutionary lineages, culminating in humans.

CRP is structurally a pentameric molecule composed of identical monomers. There are three types of CRP: monomeric CRP (mCRP), non-native pentameric CRP, and native pentameric CRP (native CRP). For host defence, both native and non-native CRP perform ligand-recognition tasks. Any pentameric CRP will dissociate into ligand-bound mCRP after binding to a ligand. If ligand-bound mCRP possesses the same proinflammatory properties as free mCRP, which have been demonstrated *in vitro*, then both mCRP and the associated ligand must be eliminated from the site of inflammation. Pentameric CRP decreases the production of foam cells and the proinflammatory effects of atherogenic low-density lipoprotein (LDL) once it has been linked to atherogenic LDL. In a mouse model of atherosclerosis, it has been discovered that a CRP mutant, also known as non-native CRP, is atheroprotective. A medication that can only decrease cholesterol levels and not CRP levels, unlike statins, should thus be created. It is plausible that non-native CRP might be protective against all inflammatory conditions in which host proteins turn pathogenic because non-native CRP has been demonstrated to bind to all types of defective proteins in general. It would be preferable to use a small-molecule drug to target CRP with the aim of changing the conformation of endogenous native CRP rather than recombinant non-native CRP as a biologic to treat diseases brought on by pathogenic proteins like oxidized LDL if research using transgenic mice demonstrates that non-native CRP is advantageous for the host (48).

CRP is generated by the liver when there is inflammation, and atherosclerosis is an inflammatory cardiovascular disease. A putative function for CRP in atherosclerosis is suggested by the co-localization of CRP and low-density lipoproteins (LDL) at atherosclerotic lesions. If the phosphocholine groups in LDL are not accessible, CRP does not interact with LDL; it only interacts with molecules containing phosphocholine. However, if CRP’s natural conformation is changed, it can attach to LDL without exposing phosphocholine groups. Site-directed mutagenesis produced the CRP mutant F66A/T76Y/E81A, which does not bind to phosphocholine. It was shown that this mutant CRP could attach to atherogenic LDL without undergoing any further conformational changes. A CRP mutant can bind to atherogenic LDL, is reported to have atheroprotective role in a murine model of atherosclerosis. Mice given mutant CRP treatment for 9 weeks had atherosclerotic lesions in their aortas that were 40% smaller in size than lesions in untreated mice’s aortas. Thus, mutant CRP gave protection against atherosclerosis, demonstrating that a structural change brought on by local inflammation in wild-type CRP is necessary for CRP to limit the growth of atherosclerosis (49).

In the acute phase of the reaction, CRP levels in the serum significantly rise. Maximum CRP expression is seen in cells treated with both IL-6 and IL-1 in human hepatoma Hep3B cells. A synergistic interaction between IL-1 and IL-6 causes transcription of the CRP gene to be induced. Four consensus signal transducer and activator of transcription (STAT3)-binding sites located at positions 72, 108, 134, and 164 on the CRP promoter regulates the function of IL-6-activated transcription factor STAT3. Prior research has demonstrated that STAT3 binds to the location at 108 and activates CRP expression. Additionally, STAT3 bound to the other three sites, and several STAT3-containing complexes developed at each site, indicating the inclusion of other transcription factors and STAT3 isoforms in the complexes. Although the synergy between IL-6 and IL-1 was unaffected by the mutations, CRP expression was lowered in response to IL-6 and IL-1 treatment when the STAT3 sites at positions 108, 134, or 164 were altered. Mutagenesis was unable to be used to study the STAT3 location at position 72. In Hep3B cells, IL-6 activated two STAT3 isoforms. The isoforms of STAT3 are STAT3α and STAT3β, where the former has both DNA-binding domain and a transactivation domain whereas the latter contains only the DNA-binding domain (50).

In hepatocytes, the promoter of human CRP lies 37.7 kb upstream of a constitutively active enhancer (E1). We demonstrate that E1 is enriched in STAT3- and C/EBP-binding sites and is required for the entire induction of human CRP during the acute phase utilizing chromatin immunoprecipitation, luciferase reporter assay, *in situ* genetic modification, CRISPRi, and CRISPRa. Furthermore, by examining the activities of E1-promoter hybrids and the related epigenetic alterations, we show that E1 coordinates with the promoter of CRP to control its variable expression across tissues and species. These findings thus point to an intriguing form of molecular evolution in which expression-changing mutations in distal regulatory elements start a process of functional selection that involves interaction between distal/proximal regulatory alterations and activity-changing coding mutations (51).

CRP is a pentraxin with characteristics like those of a pattern recognition receptor. The *in vivo* activities of CRP and its involvement in health and illness, although being widely utilized as a clinical measure of inflammation, remain largely unestablished. This is partially explained by the fact that the expression patterns of CRP in mice and rats are radically different from one another. This raises questions regarding whether these activities of CRP are crucial and preserved across species, as well as how these model animals should be treated to investigate the *in vivo* effects of human CRP. The enhanced model architecture will help define the pathophysiological functions of CRP and aid in the creation of fresh CRP-targeting tactics (52).

In conclusion, almost all phylogenetic representatives have shown over the past several decades that CRP has a variety of functions, and it continues to be an intriguing and perplexing protein for animal survival. Further research will shed light on CRP’s vital allies in the fight for survival of animals lacking an acquired immune response and will emphasize the protein’s flexibility in influencing several survival pathways.

## Author contributions

SB and CM both contributed to writing the manuscript. All authors contributed to the article and approved the submitted version.
